# Incidence and Risk Factors of Developing a Dysrhythmia After Blunt Thoracic Trauma

**DOI:** 10.3390/jcm14176253

**Published:** 2025-09-04

**Authors:** Jessica Jowers, Kevin Van Derveer, Katherine Moore, Nathaniel Harshaw, Julie M. Reichert, Hannah Karr, Urhum Khaliq, David J. Cziperle, Lindsey L. Perea

**Affiliations:** 1 Department of Surgery, Philadelphia College of Osteopathic Medicine, 4170 City Avenue, Philadelphia, PA 19131, USA; jj1164@pcom.edu (J.J.); kd3030@pcom.edu (K.V.D.); urhumkh@pcom.edu (U.K.); 2 Department of Surgery, Division of Trauma and Acute Care Surgery, Penn Medicine Lancaster General Health, 555 N Duke Street, Lancaster, PA 17604, USA; katemoore130@gmail.com (K.M.); nate.harshaw@gmail.com (N.H.); julie.reichert@pennmedicine.upenn.edu (J.M.R.); 3 Philadelphia College of Osteopathic Medicine, 4170 City Avenue, Philadelphia, PA 19131, USA; hk9458@pcom.edu; 4 Department of Surgery, Division of Thoracic Surgery, Penn Medicine Lancaster General Health, 555 N Duke Street, Lancaster, PA 17604, USA; david.cziperle@pennmedicine.upenn.edu

**Keywords:** dysrhythmia, thoracic, trauma, blunt cardiac injury, thoracic trauma, blunt thoracic trauma

## Abstract

**Background/Objectives**: The incidence of dysrhythmia after blunt thoracic trauma varies in the literature from 8–75%, and the complication rate from these dysrhythmias is not well studied. The aims of this study are to (1) identify the incidence of dysrhythmia following blunt thoracic trauma, (2) identify risk factors associated with developing a dysrhythmia, and (3) identify the incidence of cardiac intervention after developing a dysrhythmia. We hypothesize that blunt thoracic trauma may result in post-injury dysrhythmias. **Methods**: This is a retrospective review of trauma patients ≥ 18 years with a blunt mechanism of injury at a Level 1 Trauma Center from 1/2010 to 3/2022. Patients were included if they had one of the following: rib fracture, sternal fracture, chest wall contusion, pneumothorax, hemothorax, chest pain, chest wall deformity, or chest wall crepitus. Patients were excluded if they had an Abbreviated Injury Scale Chest = 0 or if they had a pre-existing dysrhythmia. Univariate, multivariate, and multivariable statistical analyses were performed. **Results**: In total, 2943 patients met inclusion criteria. In total, 574 (19.5%) developed a dysrhythmia; 100 (17.4%) required a new antiarrhythmic at discharge. Patients who developed a dysrhythmia had a nearly two times greater likelihood of requiring cardiac intervention than those without a dysrhythmia (AOR: 1.79; *p* = 0.004). Additional risk factors for requiring cardiac intervention included Injury Severity Score (ISS) 16–25 and >25 (*p* < 0.001). **Conclusions**: The incidence of dysrhythmia after blunt thoracic injury is 19.5% at our level I trauma center. Based on our study, patients that were older, had an ISS > 25, had a history of previous cardiac disease, or required > 5 units of blood products were at an increased risk of developing a dysrhythmia following trauma. As such, future consideration should be given to extended guidelines in monitoring these vulnerable patients.

## 1. Introduction

Blunt thoracic trauma is the second most common cause of death after head injury, following motor vehicle collisions, and accounts for greater than 15% of all trauma admissions worldwide. Most often, blunt thoracic trauma occurs following a high-speed sudden deceleration with direct impact to the anterior chest [1]. Visceral injuries associated with blunt thoracic trauma include cardiac contusion and injury to the ascending aorta, innominate arteries, and thoracic vertebrae. Sternal fractures, another product of blunt thoracic injury, can cause additional injuries to the right ventricle. Blunt force exerted on the heart by the sternum can also increase intraventricular pressure, which can damage or rupture cardiac valves [2]. Although many injuries occur from blunt thoracic trauma, this paper focuses on potential cardiac injury leading to dysrhythmia and further cardiac complications, whether caused by the dysrhythmia or blunt injury.

The Eastern Association for the Surgery of Trauma (EAST) guidelines currently state that all patients with suspected blunt thoracic trauma should have an EKG (Level I recommendation) and cardiac troponin (Level II recommendation) on admission [3]. Although this is the current standard of care, there are studies reporting low sensitivity and specificity of EKG and cardiac enzymes. Some even suggest they have no correlation to clinical outcomes [4,5]. Additionally, in multiple case reports, there were reported deaths due to late dysrhythmia after blunt thoracic trauma, despite normal admission of EKG and cardiac enzymes [6,7]. These cases were confirmed on autopsy to have myocardial contusion [6].

Given that EKG and cardiac enzymes do not identify all patients at risk of developing a cardiac dysrhythmia, it would be valuable to identify additional risk factors for developing a dysrhythmia. The incidence of a dysrhythmia after blunt thoracic trauma varies in the literature from 8–75%, and the complication rate from these dysrhythmias is unknown [2]. Current practice focuses on monitoring potential cardiac symptoms and treating cardiac complications as they arise. If specific patients could be identified as considerable risk for dysrhythmia and subsequent cardiac complications based on age, gender, comorbidities, mechanism of injury, and injury severity, then focus could be directed toward prevention. The aims of this study are to (1) identify the incidence of dysrhythmia following blunt thoracic trauma at a Level I Trauma Center, (2) identify risk factors associated with developing a dysrhythmia and subsequent complications, and (3) identify the incidence of cardiac intervention after developing a dysrhythmia. The data will be used to help identify specific populations at a higher risk of developing a dysrhythmia that could potentially benefit from preventative treatment on admission. We hypothesized that blunt thoracic trauma may result in post-injury dysrhythmias that subsequently undergo intervention.

## 2. Materials and Methods

Following approval from our Institutional Review Board, we performed a retrospective review utilizing data from our trauma registry and medical records from January 2010 through March 2022 at our Level I Trauma Center. All patients ≥ 18 years presenting as a trauma activation to the trauma service at our facility with a blunt mechanism of injury to the thorax were included in this analysis. Patients were included if they had one or more of the following: rib fracture(s), sternal fracture, chest wall contusion, pneumothorax, hemothorax, chest pain, chest wall deformity, and/or chest wall crepitus. Patients were excluded if they had an Abbreviated Injury Scale (AIS) Chest of zero or a pre-existing dysrhythmia. The primary outcome of this study was the development of a dysrhythmia. Secondary outcomes included unplanned intensive care unit (ICU) admission, pulmonary embolism (PE), and cerebrovascular accident (CVA).

Analyses were conducted in the form of Student’s t-tests and Mann–Whitney U tests for continuous variables and chi-squared tests and Fisher’s exact tests for categorical variables. These analyses were performed to compare unadjusted baseline demographics and clinical outcomes between patients who developed a dysrhythmia following a blunt mechanism of injury to the thorax versus those who did not. In our study, a dysrhythmia was defined as any heart rhythm that deviated from normal sinus rhythm (i.e., sinus tachycardia, sinus bradycardia, narrow and wide complex tachycardia, ventricular fibrillation, supraventricular tachycardia (SVT), atrial fibrillation (AF)/flutter, atrioventricular (AV) block, Torsades de pointes, premature atrial and ventricular contractions, pulseless electrical activity, and asystole). For non-normally distributed variables, median (Interquartile Range [IQR]) were used, whereas for normally distributed variables mean (standard deviation [SD]) were used. Analyzed variables included age, sex, race, Injury Severity Score (ISS), mechanism of injury (MOI), units of blood products received, and complications following a dysrhythmia. Development of a dysrhythmia was defined as an abnormal cardiac rhythm as observed on cardiac telemetry or EKG. Cardiac intervention was defined as cardiac catheterization, pacemaker implementation, cardioversion, defibrillation, stent placement, thoracotomy, and cardiopulmonary resuscitation (CPR). Missing data were excluded from this analysis. All statistical analyses were performed using Stata/IC, version 16.0. A *p*-value < 0.05 was considered statistically significant. A confidence interval of 95% was used.

## 3. Results

A total of 2943 patients met inclusion criteria and were included in this analysis. Of these patients, 574 (19.5%) developed a dysrhythmia following blunt trauma to the thorax. Sinus tachycardia, often a benign dysrhythmia, was included and accounted for 11.5% (*n* = 66) of dysrhythmias in our dataset. The total patient population contained more males (*n* = 1899 [64.55]) than females (*n* = 1044 [35.5%], *p* ≤ 0.001). All patients presented with a blunt mechanism of injury; 1327 (45.1%) were involved in a motor vehicle collision (MVC) and 346 (11.8%) were involved in a motorcycle collision (MCC). Within this patient population, 2749 (93.5%) patients were White/Caucasian and 136 (4.6%) patients were Black/African American. Two hundred and one (6.8%) patients included in this analysis died due to their injuries. Demographic and injury-specific characteristics of patients who developed a dysrhythmia are displayed in Table 1.

Table 1 also displays the breakdown of the mechanism of injury in patients with and without a dysrhythmia. MVC was the most common MOI for patients who developed a dysrhythmia (43.6%), followed by fall (34.7%) and MCC (10.3%). The remaining mechanisms of injury accounted for less than 11.4% of all dysrhythmias. We conducted a subgroup analysis of patients aged >64 and found that geriatric patients had the greatest incidence of dysrhythmia following a fall (45.3%) or MVC (43.6%), with remaining mechanisms causing 11.1% of dysrhythmias. Table 2 displays the mechanism of injury and associated rates of dysrhythmia for geriatric patients.

Patients with a previously noted cardiac history were more likely to develop a dysrhythmia than those without a cardiac history [History: 347 (60.56%), No History: 226 (58.6%), *p* < 0.001]). This was observed in patients with a past medical history of hypertension, coronary artery disease, aortic stenosis, valve replacement, and congestive heart failure. Additionally, a significant relationship was observed between the development of a dysrhythmia and the need for cardiac intervention as significantly fewer patients who underwent cardiac intervention had dysrhythmia (Dysrhythmia (D): 64 [33.68%], No Dysrhythmia (ND): 126 [66.32%], *p* < 0.001).

In the entire population, 190 patients required cardiac intervention. Most patients undergoing an intervention underwent a cardiac procedure apart from cardiac catheterization, pacemaker placement, cardioversion, pacing/defibrillation, stent placement, or open-heart surgery (*n* = 168 [88.4%]). Only a third (*n* = 64 [33.7%]) of patients undergoing intervention had a dysrhythmia identified during their hospitalization. When looking at cardiac interventions by MOI, cardiac catheterization occurred in 50% (*n* = 7) of patients injured by MVC, followed by 36% for falls (*n* = 5). Cardiac catheterization was performed in one MCC patient (7.14%) and one patient injured by an animal (7.14%). Seven patients had pacemaker implantation, 85.71% (*n* = 6) of which were for patients who sustained a fall. Cardioversion was performed in three patients injured via MVC and one patient injured via fall. Pacing was performed in only one patient and that patient was involved in an MVC. Defibrillation was performed in 12 patients with 75% (*n* = 8) of these patients injured via MVC. Cardioversion was performed for three MVC patients and one patient injured via MCC. Additionally, one patient underwent open heart surgery, and one patient had a stent placement; both these patients were injured in an MVC.

In the entire study population, a total of 2314 (78.6%) patients received an EKG. The most frequently recorded dysrhythmias among our study population included bradycardia (*n* = 181, 24.4%), premature ventricular contraction (*n* = 133, 17.9%), premature atrial contraction (*n* = 116, 15.7%), type 1 AV block (*n* = 116, 15.7%), and AF (*n* = 74, 10.0%). There was a significant association between the development of a dysrhythmia and the administration of an antiarrhythmic medication during admission (administered anti-arrhythmic: 274 (47.7%), no administered anti-arrhythmic: 300 (52.3%), *p* < 0.001). Patients with atrial flutter (*p* < 0.001), AF (*p* < 0.001), supraventricular tachycardia (*p* < 0.001), and wide complex tachycardia (*p* < 0.001) were significantly more likely to receive anti-arrhythmic medications in-hospital. Additionally, those with documented dysrhythmia during their admission were significantly more likely to have had a previous pacemaker placement (*p* < 0.001).

In a subgroup analysis of geriatric patients, a total of 606 (20.6%) patients had elevated troponin at some point during their admission, with 365 (12.4%) patients presenting with elevated troponins. Of the patients with elevated troponins during their admission, 223 (36.8%) had a documented dysrhythmia (*p* < 0.001). Documented dysrhythmia was determined based on the patient’s EKG reads, with the mean number of days from admission to the first abnormal EKG being 1.63 (3.28 days). Over 25% of our study population had a dysrhythmia at the time of their admission, and the greatest number of days from admission to the development of a dysrhythmia was 15 days.

The type of cardiac interventions performed and the number of patients that underwent intervention based on the presence or absence of a dysrhythmia on arrival are shown in Table 3. The type of cardiac history in patients that present with and without a dysrhythmia are also illustrated in Table 3. Multivariable analyses showing risk factors for the development of dysrhythmia, undergoing cardiac intervention, and readmission for cardiac complications following blunt cardiac injury are shown in Table 4. The results of the multivariate analyses examining risk factors for developing in-hospital complications, such as PE, deep vein thrombosis (DVT), myocardial infarction (MI), CVA, unplanned ICU admission, unplanned intubation, and unplanned return to the operating room, are shown in Table 5.

When stratifying by AIS chest, a multivariable logistic regression examining risk for developing a dysrhythmia found that in those with an AIS = 4 (more severe chest injury), patients with a cardiac history (AOR: 2.75 [95% CI: 1.04–7.24], *p*= 0.041) and patients receiving greater than 10 units of blood products (AOR: 4.05 [95% CI: 1.29–12.71], *p* = 0.017) had a significantly greater risk of developing dysrhythmia. A similarly parsed-out logistic regression examining the risk of undergoing cardiac intervention found that in those with a more severe chest injury and receiving greater than 10 units of blood products also had a significantly greater risk of undergoing a cardiac intervention (AOR: 7.88 [95% CI: 2.18–28.50], *p* = 0.002).

## 4. Discussion

In our study, we found a 19.5% incidence of dysrhythmia following blunt thoracic trauma. A majority of those who developed a dysrhythmia presented with normal EKG and troponin at admission. Those who received greater than five units of packed red blood cells (pRBC) and/or had an ISS > 16 were at an increased risk of developing a dysrhythmia. As the ISS increased (i.e., more severely injured patients) so did the risk of undergoing cardiac intervention. Therefore, our hypothesis supported that blunt thoracic trauma may result in tissue damage that causes post-injury dysrhythmias requiring intervention.

Myocardial contusion (tissue injury) is seen in 60–100% of blunt thoracic traumas on autopsy [2]. It is the second most common injury in blunt thoracic trauma leading to death [1]. This is different from myocardial concussion, which is abnormal wall motion without tissue injury. Myocardial concussion is not benign; however, as up to 24% of autopsies from thoracic trauma reveal myocardial concussion [1]. Despite the prevalence of myocardial contusion and concussion in blunt thoracic trauma, there is no well-established method to clinically identify either condition. In a 2004 prospective study, patients with blunt thoracic trauma were followed with serial cardiac enzymes. Thirty-four percent of patients had elevated cardiac enzymes. Of these, 25% developed a dysrhythmia. The remaining patients without elevated cardiac enzymes remained asymptomatic throughout their hospital stay [8]. Therefore, it was concluded from this study that patients with elevated cardiac enzymes had known myocardial contusion. Similarly, a meta-analysis by Maenza et al. attempted to identify patients with blunt cardiac injury who were at risk of developing cardiac complications. In total, 25 prospective and 16 retrospective studies were examined and concluded that abnormal electrocardiogram (EKG) and elevated creatine kinase-MB (CK-MB) obtained in the emergency room correlated with an increased risk of cardiac complications requiring treatment [9]. These findings suggest that cardiac evaluation with EKG and enzyme assays may be helpful in identifying those with cardiac injuries requiring monitoring and treatment. However, as can be seen in the data, there is still a significant portion of the population which suffers from cardiac complications without presenting initially with dysrhythmia. This suggests that further studies should be conducted to elucidate potential biochemical markers or methods to identify at-risk patients.

Only 37% of patients with abnormal troponins developed a dysrhythmia in our study. The remaining 63% of patients who developed a dysrhythmia had normal cardiac enzymes during their admission. In patients that developed a dysrhythmia, the time to an abnormal EKG was ~1.5 days, and the greatest time to an abnormal EKG was 15 days. Therefore, most patients who developed a dysrhythmia had a normal EKG and normal cardiac enzymes on admission. This is consistent with several case reports of patients developing dysrhythmias after blunt thoracic trauma, regardless of normal admission cardiac enzymes and EKG [6,7]. These findings suggest that 24 h is not an adequate duration for monitoring because not all dysrhythmias were identified in this timeframe. A 2020 case report in the American Journal of Forensic Medicine and Pathology, examined a dysrhythmia after blunt thoracic trauma that did not appear on EKG until post-injury day fifteen [7]. Patient mortality was a result of acute mitral insufficiency and left ventricular failure. Autopsy results reveal cardiac contusion leading to mitral insufficiency. Although a dysrhythmia does not necessarily lead to mortality, it can often be associated with more serious cardiac conditions that do lead to mortality. Our data support current guidelines suggesting continuous cardiac monitoring for 24–48 h in all patients with high suspicion of cardiac injury. However, because not all patients initially present with elevated troponin or abnormal EKG, it may be reasonable to expand the patient population that we consider as an elevated risk.

MVCs resulted in the largest percentage of patients undergoing a cardiac intervention. Regardless of the speed at which an MVC occurs, there is a large energy transfer due to the mass of the vehicle. This creates a large energy transfer to the occupants of the vehicle which may contribute to a larger percentage of blunt cardiac injury regardless of direct thoracic trauma [10]. These data may suggest that these patients are more likely to experience a dysrhythmia during their hospitalization and therefore may require a longer period of monitoring because they have experienced a more “high-risk” mechanism. Most patients in our study that underwent cardiac intervention were involved in MVC, suggesting that this high energy mechanism can induce injury despite a lack of evidence for direct thoracic trauma.

Our study also found that older patients had increased odds of developing dysrhythmia and, subsequently, a cardiac complication. This finding in our study was different from a large matched case-control study in 2007 by Ismailov et al. In this study, they evaluated exposure to thoracic trauma and the outcome of cardiac dysrhythmia. After adjusting for confounding factors, they reported that younger patients, under 50 years old, suffering cardiac injury after thoracic trauma had a four-fold increase in their risk of dysrhythmia [11]. It is possible that the cause of dysrhythmia in our study may be due to a lower physiologic “tipping point” in older individuals, as they may have less cardiac reserve in blunt thoracic injury. This could be further supported by the greater percentage of geriatric patients that developed a dysrhythmia from falls, which is considered a low energy mechanism.

Receiving more than five units of pRBC and having an ISS > 16 were independent risk factors for developing a dysrhythmia, undergoing cardiac intervention, and readmission for cardiac complications. This is likely due to a combination of greater force transfer to the thorax and hypotension leading to decreased perfusion of the already contused myocardium. Hypovolemia and orthostatic hypotension are known precipitators of AF [12,13]. It is reasonable to suggest that hypotension from hemorrhagic shock would have a similar mechanism. Future studies could investigate this further. In a study published in 2021, all trauma patients between 2007 and 2010 were identified in the Healthcare Cost and Utilization Project State Inpatient Databases for California and Florida. They found that patients with new onset AF following any trauma were at a higher risk of developing a MI, CVA, or inpatient mortality [14]. In our study, patients requiring greater than five units pRBC or with an ISS > 16 were more likely to undergo cardiac catheterization, pacemaker implantation, cardioversion, defibrillation, stent placement, thoracotomy, and/or CPR. This subset of patients also had a higher rate of AF and wide complex tachycardia. Patients with these specific dysrhythmias were also more likely to be prescribed long term antiarrhythmics at discharge. Morbidity was increased in those with a high ISS or with significant transfusion requirements, from a cardiac standpoint alone. Esme et al. evaluated trauma and injury severity score (TRISS), lung injury score (LIS), and ISS to assess the prognostic importance in blunt thoracic trauma. Like our results, they found that TRISS was an independent predictor of mortality, LIS was an independent predictor of morbidity, and ISS was an independent predictor of both hospital and ICU LOS [15].

The prevention of dysrhythmia is an area not well studied in trauma or critically ill patients. There are prevention protocols in cardiothoracic surgery that have been shown to decrease postoperative morbidity and mortality from dysrhythmias [16,17]. Multiple studies have shown significant benefits using amiodarone for pharmacologic prophylaxis to prevent AF in cardiac and thoracic surgery patients; however, this has not been studied in the blunt thoracic trauma population [16,17]. Due to the novelty of prophylactic antiarrhythmic therapy, there is no standard protocol, and caution should be used if antiarrhythmic therapy is considered. We now know the incidence of dysrhythmia following blunt thoracic trauma at our Level I Trauma Center is 19.5%. Of those, the patients in critical condition receiving a transfusion are at an even greater risk for dysrhythmia and often undergo cardiac interventions. Further investigation is necessary in this patient population to determine if a prevention protocol would translate to a decreased incidence of dysrhythmia and subsequent morbidity and mortality if utilized in the critically ill trauma patient suffering blunt thoracic injury.

This study is not without limitations. First, this is a retrospective, single-center study, and thus may not be generalizable to the public. Given the retrospective nature, it is impossible to control for all confounding factors, such as medication use (e.g., beta-blockers, etc.) or pre-existing electrolyte abnormalities which may also influence the development of a dysrhythmia. Further, distinguishing which dysrhythmias were clinically relevant was challenging due to the retrospective nature of the study and the inability to determine causality. We found 11.5% of patients with a dysrhythmia had sinus tachycardia, often a benign dysrhythmia. However, other dysrhythmias, such as PVC, which are traditionally thought to be benign were associated with bigeminy in our study, leading us to consider them clinically relevant. Additionally, we are unable to determine which EKGs were obtained for screening versus diagnostic purposes, as not all patients underwent systematic EKGs or enzyme screening. We found that 78.6% of patients in our study received an EKG, and, as these were not performed universally, there is an added layer of selection bias that needs to be considered. Future studies may obtain screening EKGs as part of the treatment algorithm. Because rhythm disturbances were detected via a combination of continuous telemetry and intermittent EKG, this may have led to variability in detection. We may have a relatively older population compared to other institutions, which may have led to our difference in outcomes regarding age compared to what has previously been published. Having an older population could also increase the number of patients with undiagnosed, pre-existing dysrhythmias, which is a confounding factor difficult to assess given the retrospective nature of this study. Further, we did not conduct any long-term follow-up on patients in this study, so we are unable to determine post-discharge morbidity and mortality, as well as readmission for cardiac-related events, which would provide a more comprehensive conclusion.

## 5. Conclusions

Blunt thoracic trauma accounts for many deaths annually, and myocardial contusion is seen in almost all autopsies following death by this mechanism. Our study demonstrates that 19.5% of patients with blunt thoracic trauma at a Level I Trauma Center developed dysrhythmia. Cardiac history, injury severity, and blood product administration were found to be significant risk factors in developing a dysrhythmia. Additionally, with increasing ISS and transfusion amount, the risk of cardiac intervention and readmission for cardiac complications increased. Further studies could focus on prevention of dysrhythmia in critically ill trauma patients, as well as those at higher risk for cardiac complications post blunt thoracic injury, given the morbidity associated with various cardiac procedures and medications.

## Figures and Tables

**Table 1:** Demographic and injury characteristics for patients with and without dysrhythmia.

	No Dysrhythmia (*n* = 2364)	Dysrhythmia (*n* = 574)	*p*-Value
Age ^a^	50 (32–65)	64 (45–78)	<0.001
Male Gender ^b^	1548 (65.5)	347 (60.5)	0.024
Mechanism of Injury ^b^			<0.001
MVC	1075 (45.47)	250 (43.55)	
MCC	286 (12.10)	59 (10.28)	
Bicycle	82 (3.47)	10 (1.74)	
Pedestrian	98 (4.15)	25 (4.36)	
Industrial	22 (0.93)	2 (0.35)	
Farming	17 (0.72)	1 (0.17)	
Off-Road Vehicle	4 (0.17)	3 (0.52)	
Sports/Recreation	17 (0.72)	4 (0.70)	
Fall	583 (24.66)	199 (34.67)	
Assault	25 (1.06)	2 (0.35)	
Buggy	46 (1.95)	5 (0.87)	
Animal	29 (1.23)	6 (1.05)	
Other	52 (2.20)	8 (1.39)	
Injury Severity Score (ISS) ^a^	13 (9–18)	14 (9–22)	<0.001
Units of Blood Products ^c^	0.74 (0.064)	2.34 (0.272)	<0.001
Vent Days ^a^	0 (0–0)	0 (0–1)	<0.001
ICU Length of Stay ^a^	0 (0–2)	1 (0–5)	<0.001
Hospital Length of Stay ^a^	3 (1–6)	5 (2–11)	<0.001
Mortality ^b^	115 (4.86)	86 (14.98)	<0.001

(^a^ Median (Interquartile Range); ^b^ *n* (% of population); ^c^ Mean ± Standard Deviation; MVC: Motor Vehicle Collision; MCC: Motorcycle Collision; ICU: Intensive Care Unit).

**Table 2:** Geriatric subgroup analysis: demographic and injury characteristics for patients with and without a dysrhythmia.

	No Dysrhythmia (*n* = 611)	Dysrhythmia (*n* = 280)	*p*-Value
Age ^a^	74 (69–81)	79 (72–85)	<0.001
Male Gender ^b^	334 (54.66)	143 (51.07)	0.318
Mechanism of Injury ^b^			0.419
MVC	275 (45.01)	122 (43.57)	
MCC	19 (3.11)	11 (3.93)	
Bicycle	16 (2.62)	5 (1.79)	
Pedestrian	24 (3.93)	6 (2.14)	
Industrial	3 (0.49)	1 (0.36)	
Farming	1 (0.16)	0 (0)	
Off-Road Vehicle	2 (0.33)	1 (0.36)	
Sports/Recreation	3 (0.49)	1 (0.36)	
Fall	235 (38.46)	127 (45.36)	
Assault	4 (0.65)	0 (0)	
Buggy	12 (1.96)	1 (0.36)	
Animal	7 (1.15)	1 (0.36)	
Other	10 (1.64)	4 (1.43)	
Injury Severity Score (ISS) ^a^	12 (9–17)	13 (9–20.5)	0.021
Units of Blood Products ^a^	0 (0–0)	0 (0–1)	<0.001
Vent Days ^c^ *	0.55 (2.69)	1.84 (5.29)	<0.001
ICU Length of Stay ^a^	1 (0–2)	1 (0–4)	<0.001
Hospital Length of Stay ^a^	3 (2–6)	5 (3–9)	<0.001
Mortality ^b^	52 (8.51)	49 (17.50)	<0.001

(^a^ Median (Interquartile Range); ^b^ *n* (% of population); ^c^ mean (standard deviation); MVC: Motor Vehicle Collision; MCC: Motorcycle Collision; ICU: Intensive Care Unit); * data were not normally distributed but are presented as mean (standard deviation) to show the difference between groups, which was not apparent as median (IQR).

**Table 3:** Past cardiac history and cardiac interventions based on initial presence of dysrhythmia.

	No Dysrhythmia (*n* = 2364)	Dysrhythmia (*n* = 574)	*p*-Value
Past Cardiac History			
Hypertension	820 (34.69)	319 (55.57)	<0.001
CAD	190 (8.04)	114 (19.86)	<0.001
STEMI	5 (0.21)	3 (0.52)	0.199
NSTEMI	33 (1.40)	16 (2.79)	0.020
Aortic Stenosis	14 (0.59)	22 (3.83)	<0.001
Mitral Valve Prolapse	5 (0.21)	3 (0.52)	0.199
Valve Replacement	9 (0.38)	11 (1.92)	<0.001
CHF	62 (2.62)	78 (13.59)	<0.001
Other	133 (5.63)	110 (19.16)	<0.001
Cardiac Intervention			
Cardiac Catheterization	5 (0.21)	9 (1.57)	<0.001
Pacemaker Implantation	3 (0.13)	4 (0.70)	0.012
Synchronized Cardioversion	1 (0.04)	3 (0.52)	0.005
Pacing	0 (0)	1 (0.17)	0.042
Defibrillation	8 (0.34)	6 (1.05)	0.027
Open Heart	0 (0)	1 (0.17)	0.042
Stent Placement	1 (0.04)	0 (0)	0.622
Other	119 (5.03)	49 (8.54)	0.001

Data presented as *n* (% of population); CAD: Coronary Artery Disease; STEMI: ST-segment elevation; myocardial infarction; NSTEMI: Non-ST-elevation myocardial infarction; CHF: Congestive Heart Failure.

**Table 4:** Multivariable analyses showing risk factors associated with dysrhythmia undergoing cardiac intervention and readmission for cardiac complication following blunt cardiac injury.

Risk Factors for Developing Dysrhythmia	Odds Ratio	95% Confidence Interval	*p*-Value
Age	1.022	1.016–1.029	<0.001
ISS Greater than 25	2.350	1.429–2.861	<0.001
GCS of 13–15	0.501	1.429–2.861	0.015
Receiving 5–10 Units of Blood Products	2.230	1.360–3.660	0.002
Receiving > 10 Units of Blood Products	1.790	1.730–5.480	<0.001
Cardiac History	1.790	1.351–2.202	<0.001
Risk Factors for Undergoing Cardiac Intervention			
Development of Dysrhythmia	1.790	1.210–2.653	0.004
Fall MOI	0.067	0.004–1.019	0.054
Greater TRISS	0.133	0.036–0.492	0.003
ISS of 16–25 (Severe)	4.050	2.470–6.652	<0.001
ISS Greater than 25 (Profound)	9.120	5.372–15.468	<0.001
Risk for Being Readmitted for Cardiac Complications			
Age	1.040	1.017–1.072	0.001
ISS of 16–25 (Severe)	2.350	0.961–5.895	0.061
ISS Greater than 25 (Profound)	7.000	1.539–31.875	0.012

(ISS: Injury Severity Score; GCS: Glasgow Come Scale; MOI: Mechanism of Injury; TRISS: Trauma and Injury Severity Score).

**Table 5:** Multivariate analyses evaluating risk factors for developing in-hospital complications.

Risk Factors for Developing In-Hospital Complications	Odds Ratio	95% Confidence Interval	*p*-Value
Pulmonary Embolism (PE)			
Development of Dysrhythmia	2.97	1.048–8.417	0.041
ISS of 16–25 (Severe)	6.54	1.700–25.255	0.006
ISS Greater than 25 (Profound)	8.56	1.673–43.850	0.010
Cardiac History	0.15	0.028–0.763	0.023
Deep Vein Thrombosis (DVT)			
ISS of 16–25 (Severe)	4.31	1.423–13.040	0.010
ISS Greater than 25 (Profound)	11.87	3.760–37.420	<0.001
Receiving 5–10 Units of Blood Products	3.86	1.390–10.740	0.010
Receiving > 10 Units of Blood Products	12.79	5.010–32.660	<0.001
Myocardial Infarction (MI)			
Age	1.07	1.020–1.120	0.006
Male Gender	3.67	1.040–13.040	0.044
Receiving 5–10 Units of Blood Products	18.20	4.600–72.020	<0.001
Cerebrovascular Accident (CVA)			
Development of Dysrhythmia	3.06	1.070–8.800	0.038
Unplanned ICU Admission			
Development of Dysrhythmia	2.39	1.510–3.800	<0.001
Undergoing Cardiac Intervention	2.23	1.130–4.410	0.021
Unplanned Intubation			
Development of Dysrhythmia	2.88	1.820–4.560	<0.001
Unplanned Return to Operating Room			
Development of Dysrhythmia	3.12	1.520–6.420	0.002

(ISS: Injury Severity Score; ICU: Intensive Care Unit).

## Data Availability

Due to privacy and ethical restrictions, data sharing is not applicable to this article.

## Abbreviations

The following abbreviations are used in this manuscript:

EKG	Electrocardiogram
CK-MB	Creatine Kinase-MB
EAST	Eastern Association for the Surgery of Trauma
AIS	Abbreviated Injury Scale
ICU	Intensive Care Unit
PE	Pulmonary Embolism
CVA	Cerebrovascular Accident
SVT	Supraventricular Tachycardia
AF	Atrial Fibrillation
AV	Atrioventricular
IQR	Interquartile Range
SD	Standard Deviation
ISS	Injury Severity Score
MOI	Mechanism of Injury
CPR	Cardiopulmonary Resuscitation
MVC	Motor Vehicle Collision
MCC	Motorcycle Collision
D	Dysrhythmia
ND	No Dysrhythmia
DVT	Deep Vein Thrombosis
MI	Myocardial Infarction
AOR	Adjusted Odds Ratio
CI	Confidence Interval
pRBC	Packed Red Blood Cells
TRISS	Trauma and Injury Severity Score
LIS	Lung Injury Score
LOS	Length of Stay
